# Supplementary figures and images for: Improving Gaussian Naive Bayes classification on imbalanced data through coordinate-based minority feature mining
Source: PeerJ Comput Sci. 2025 Jul 3;11:e3003. doi: 10.7717/peerj-cs.3003 (PMC12453872; doi:10.7717/peerj-cs.3003)

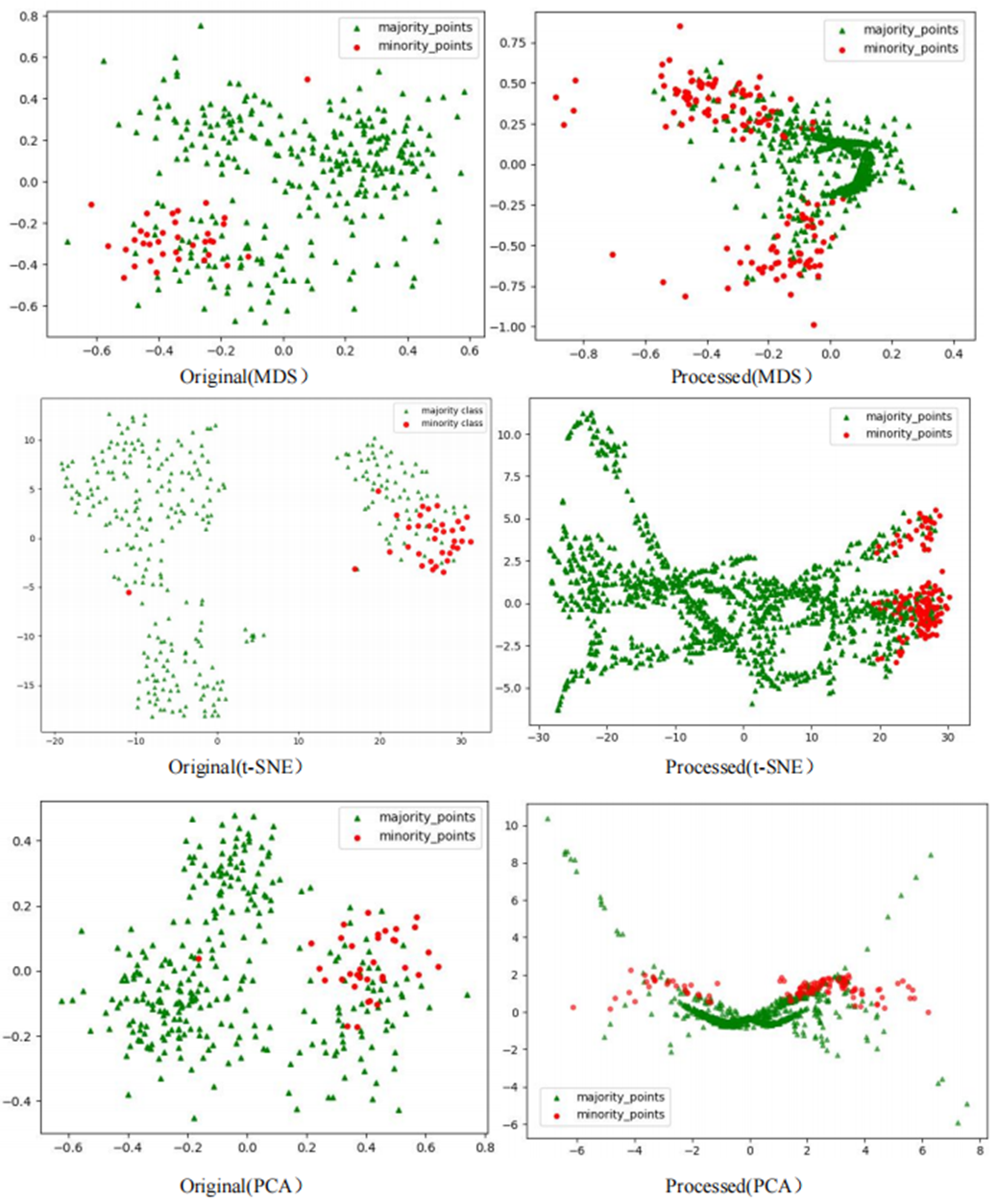

Supplement: Supplemental Information 1 [file peerj-cs-11-3003-s001.png]

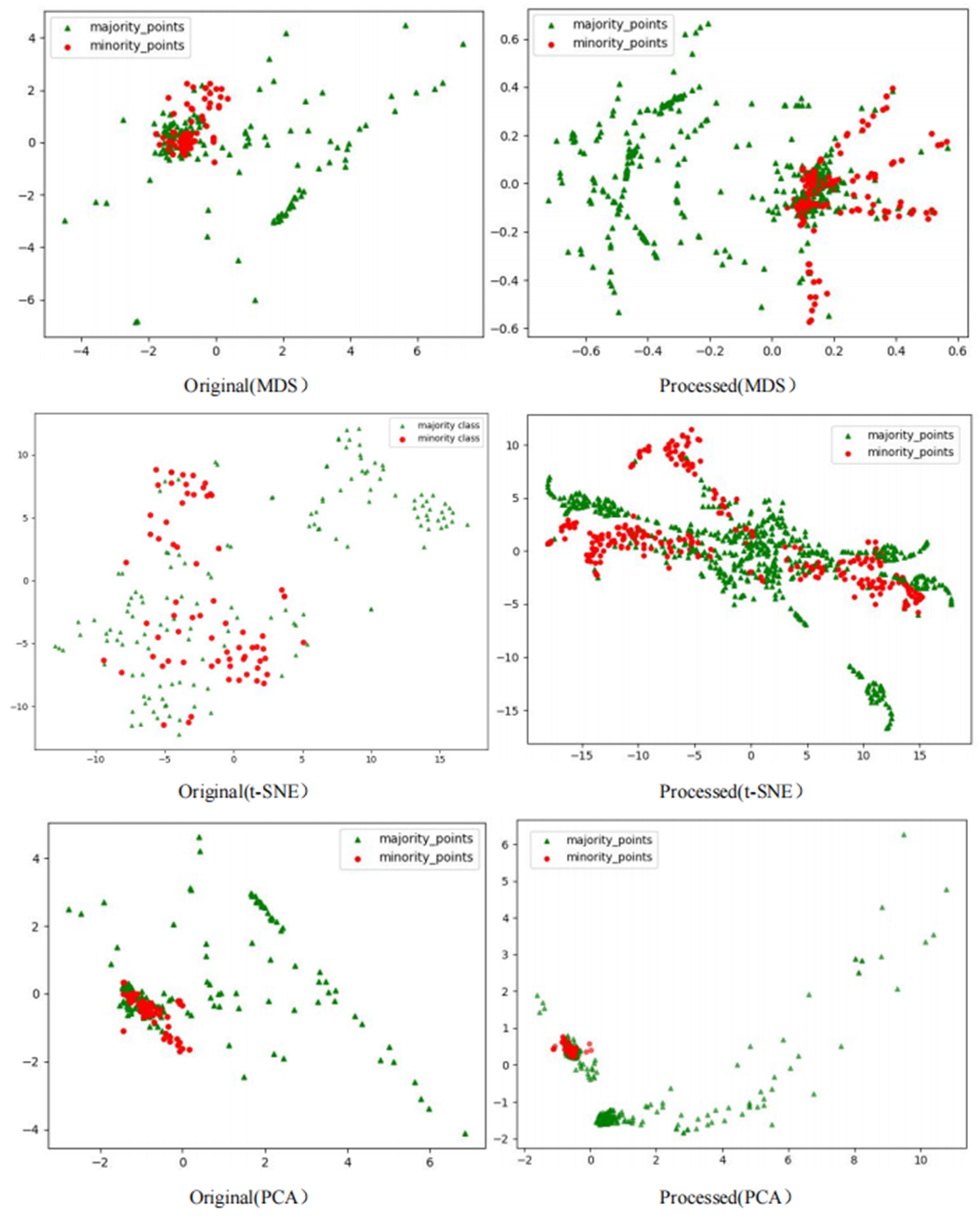

Supplement: Supplemental Information 2 [file peerj-cs-11-3003-s002.png]

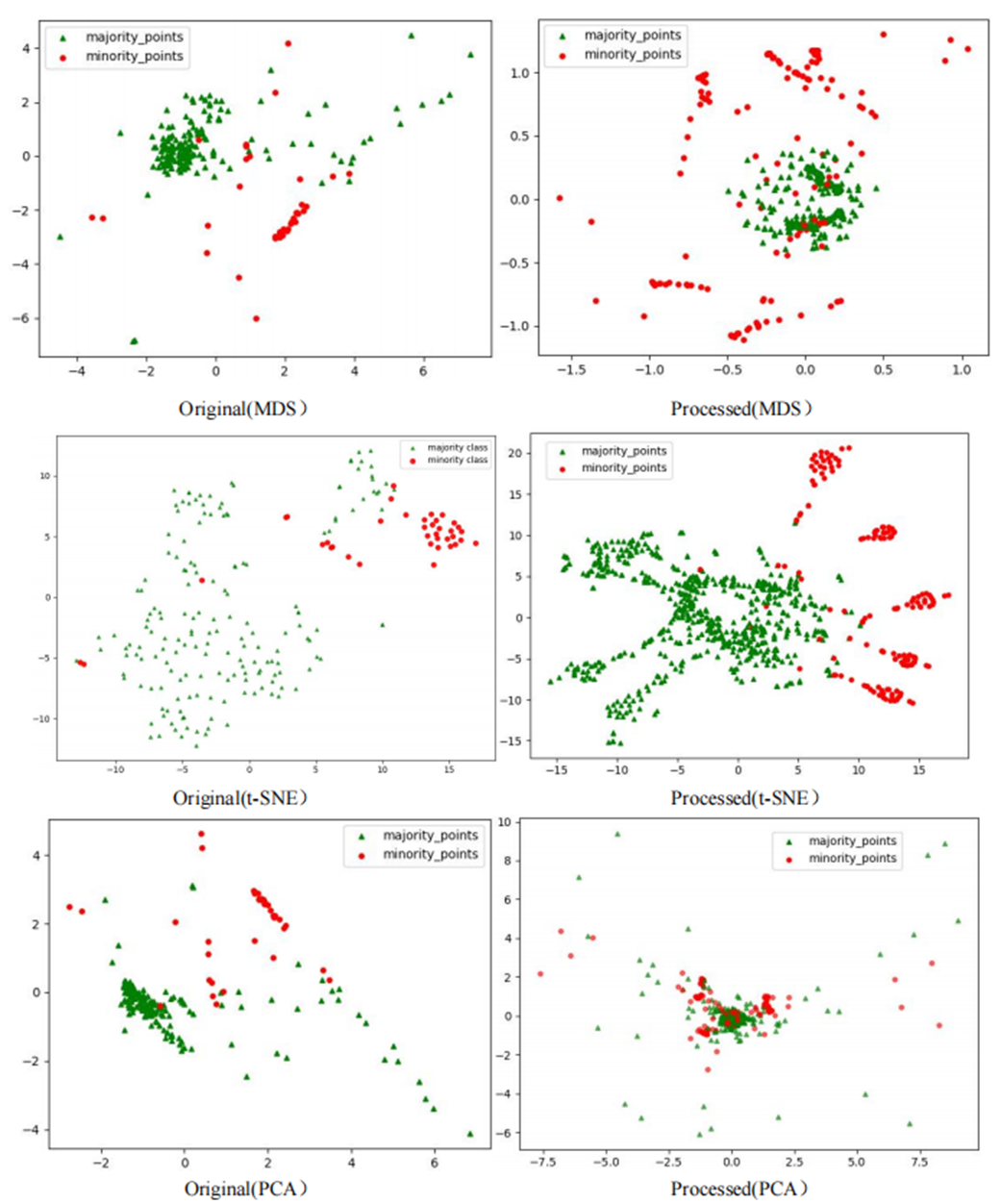

Supplement: Supplemental Information 3 [file peerj-cs-11-3003-s003.png]

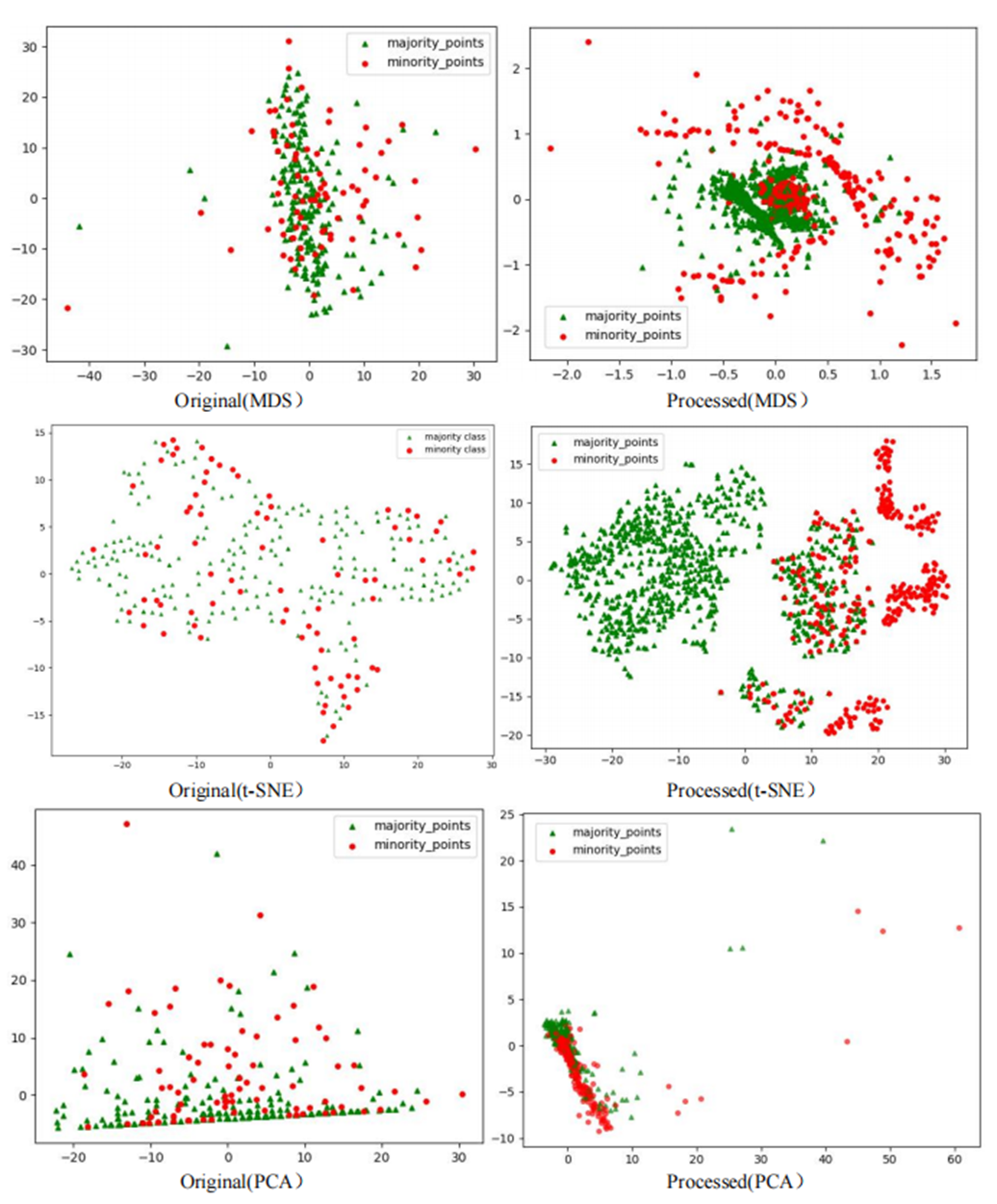

Supplement: Supplemental Information 4 [file peerj-cs-11-3003-s004.png]

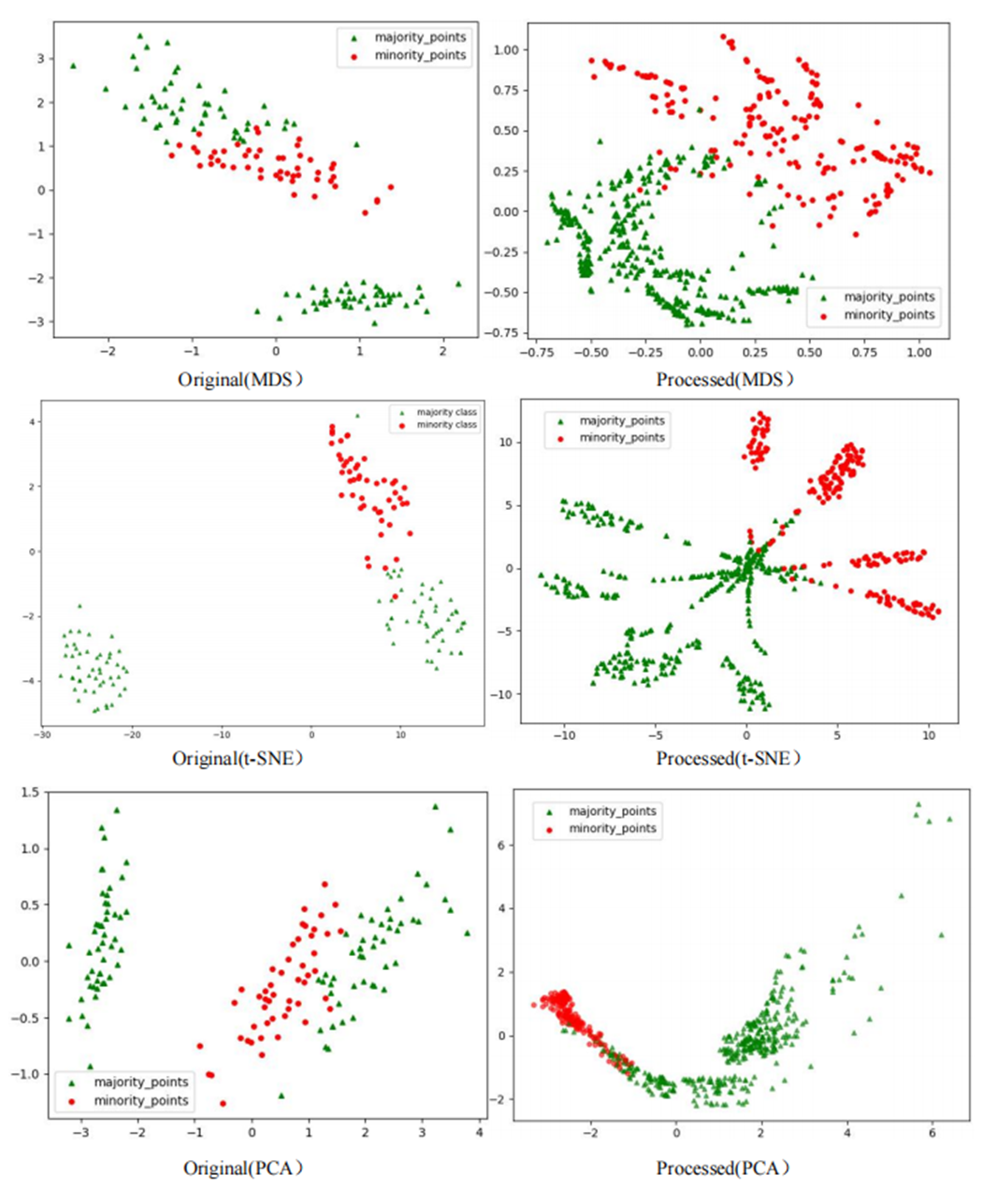

Supplement: Supplemental Information 5 [file peerj-cs-11-3003-s005.png]

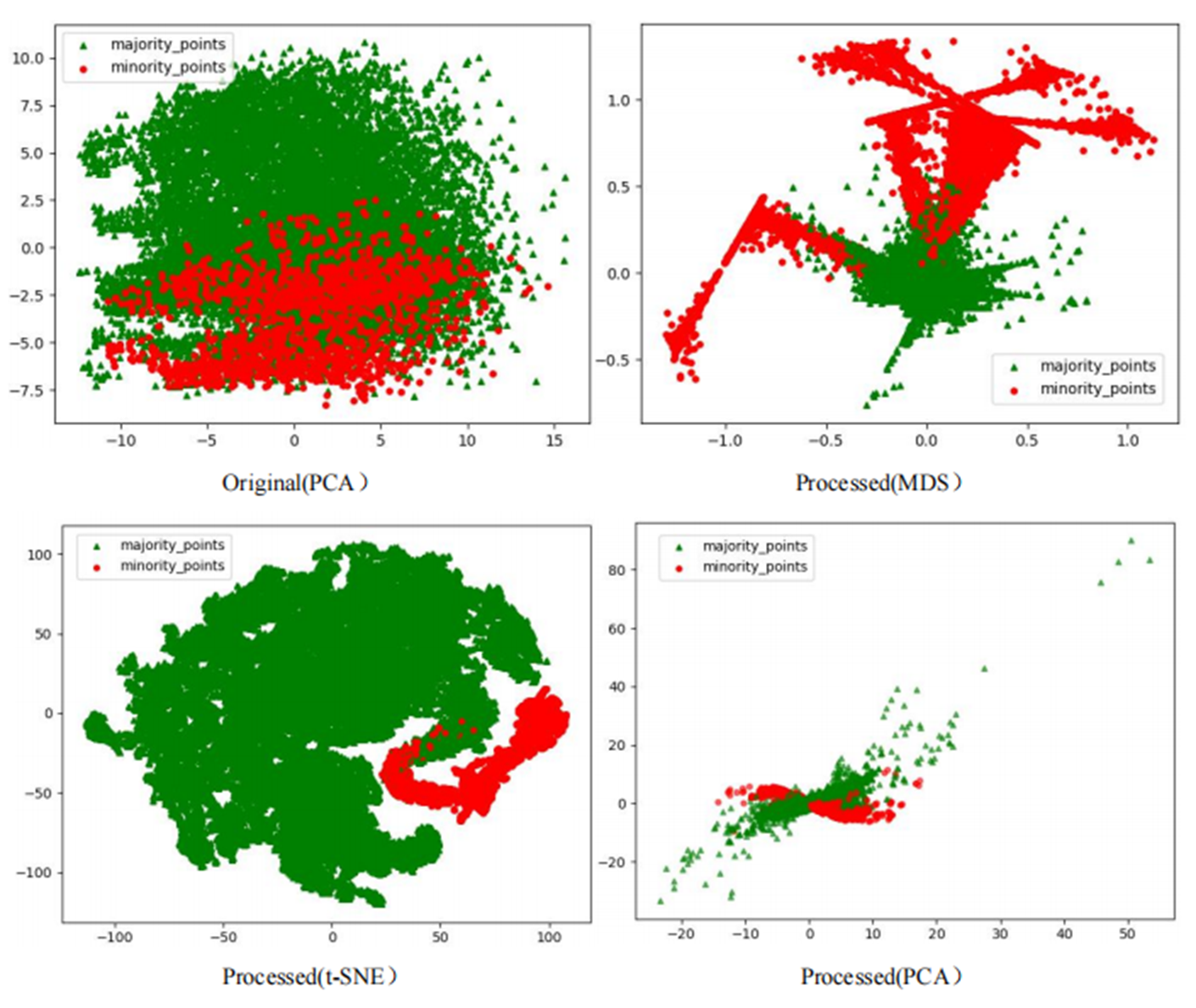

Supplement: Supplemental Information 6 [file peerj-cs-11-3003-s006.png]

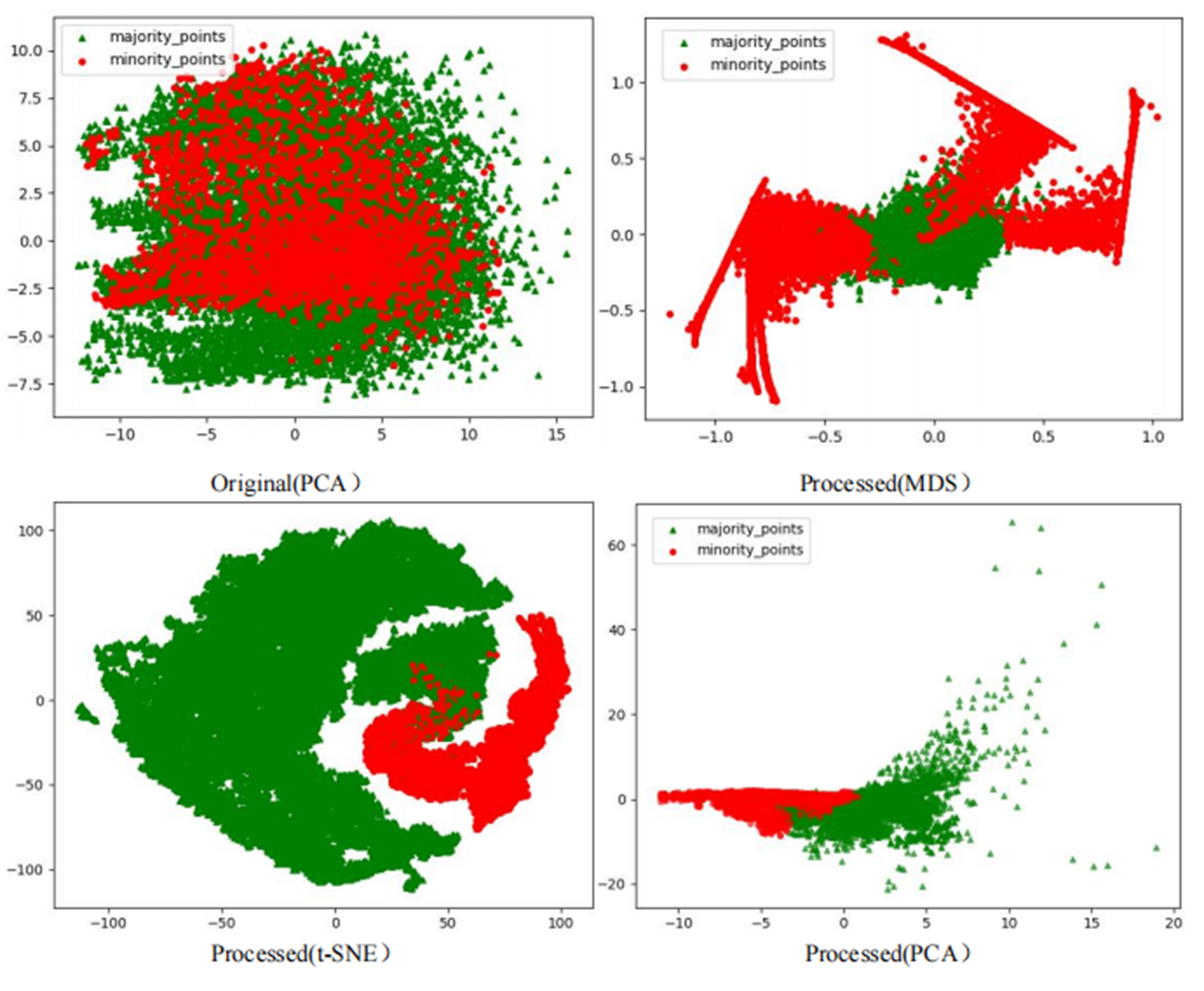

Supplement: Supplemental Information 7 [file peerj-cs-11-3003-s007.png]

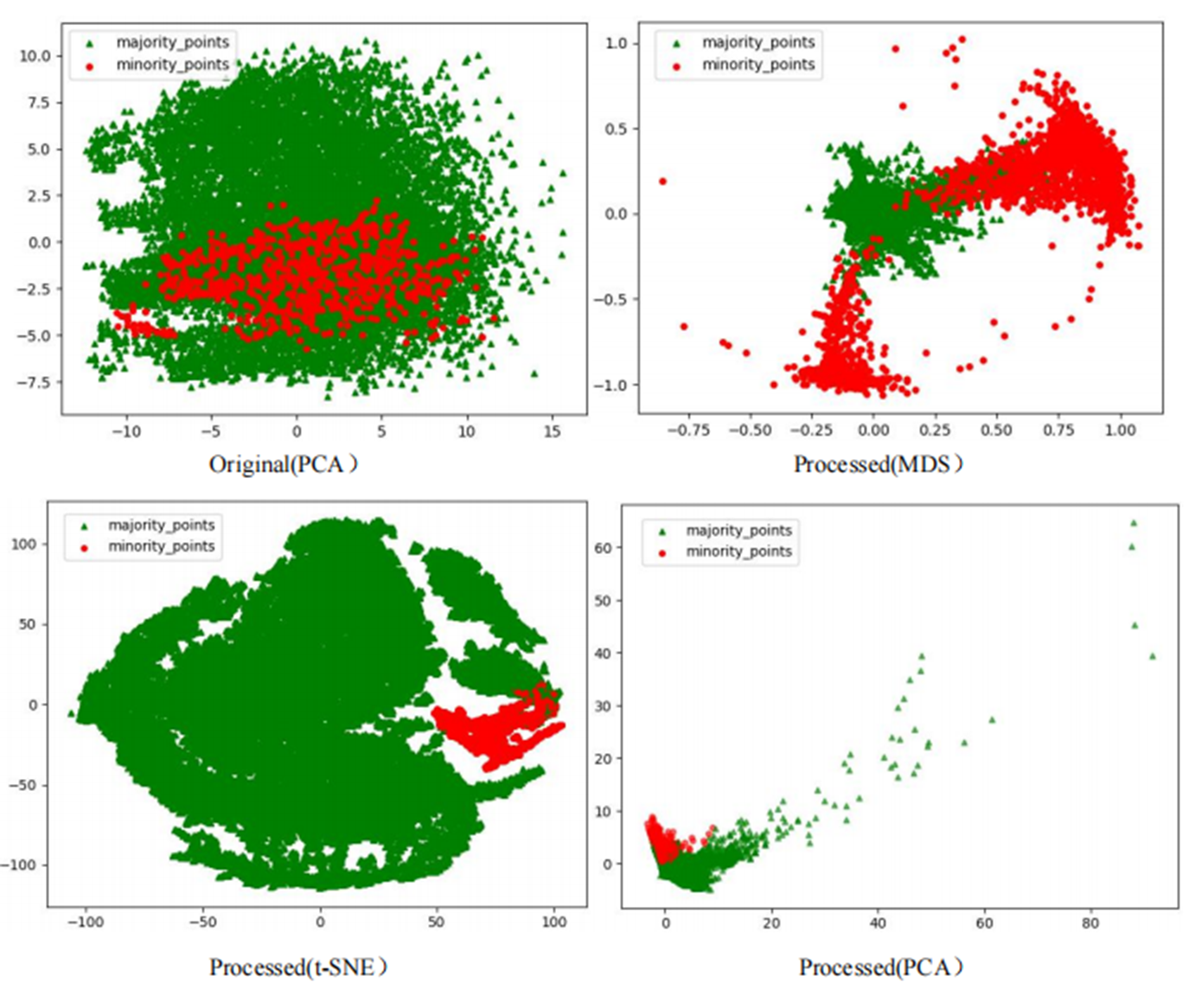

Supplement: Supplemental Information 8 [file peerj-cs-11-3003-s008.png]

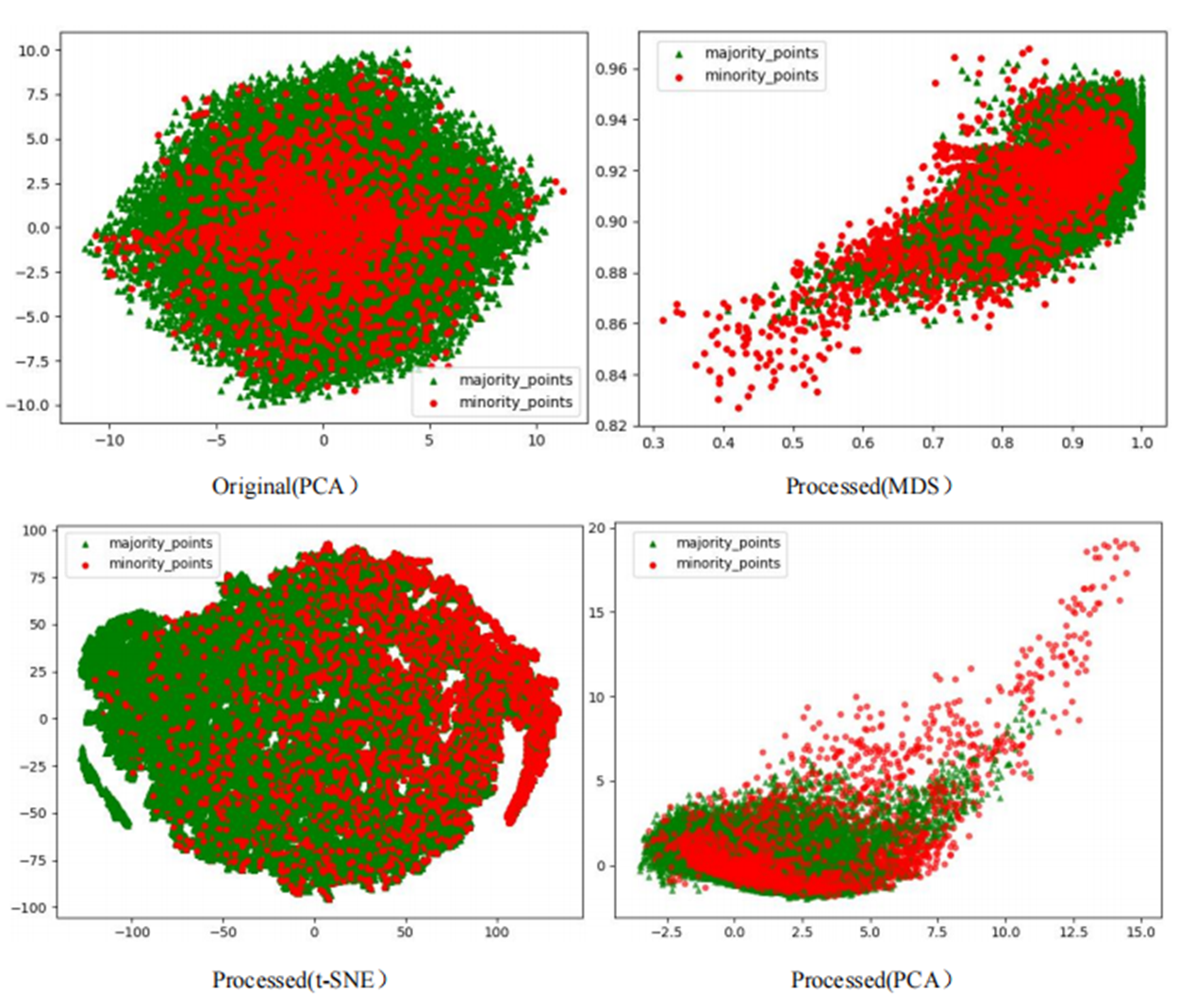

Supplement: Supplemental Information 9 [file peerj-cs-11-3003-s009.png]

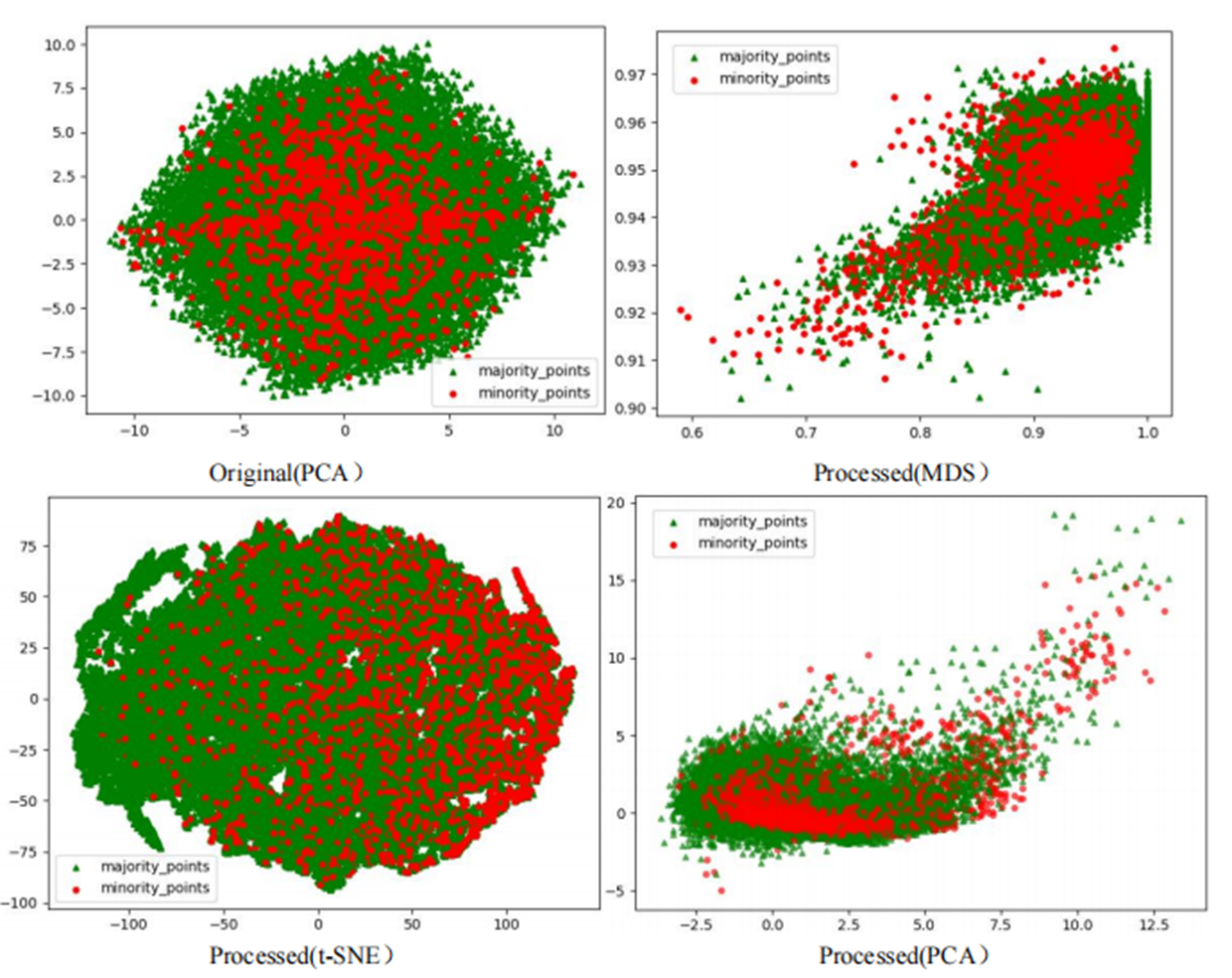

Supplement: Supplemental Information 10 [file peerj-cs-11-3003-s010.png]

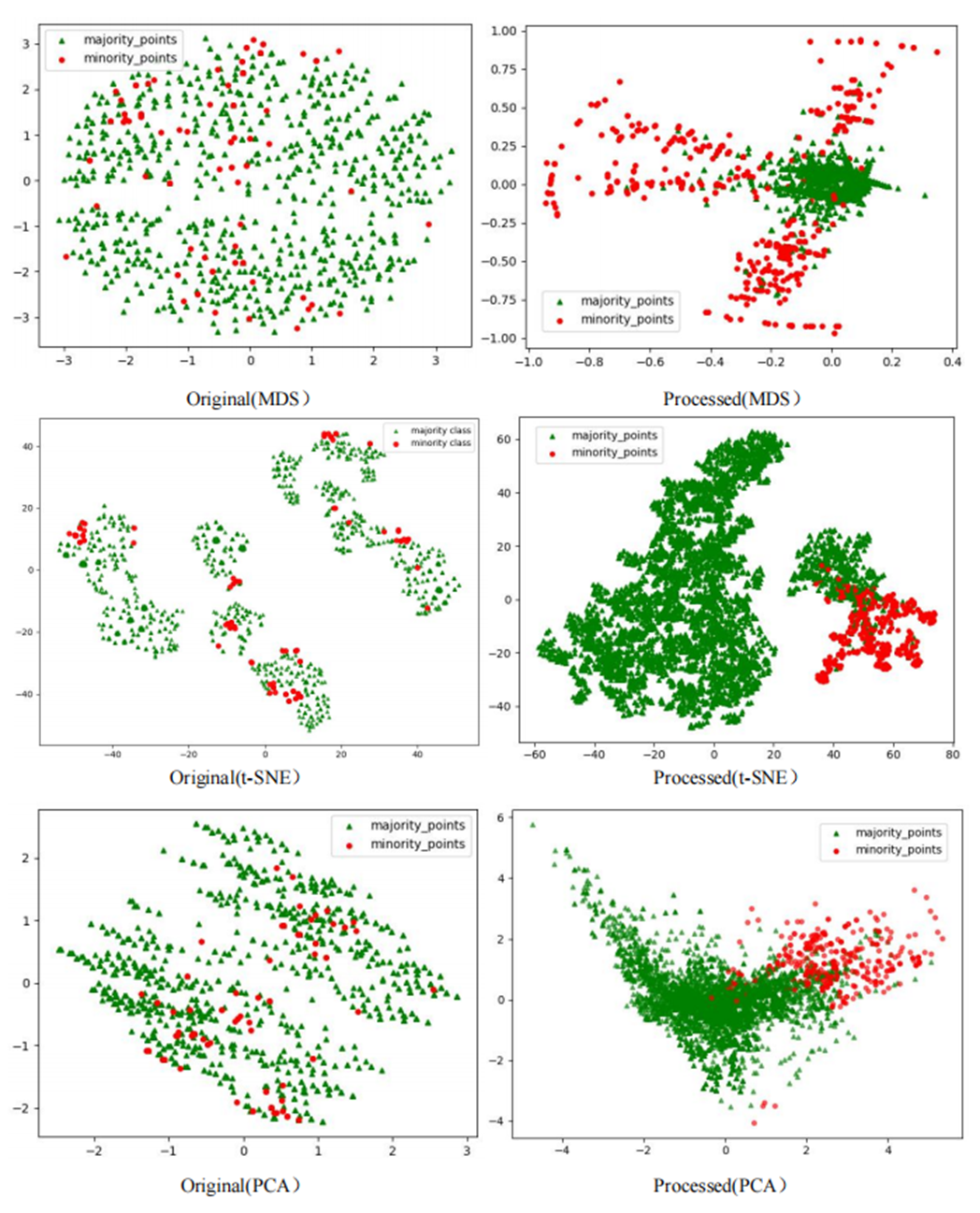

Supplement: Supplemental Information 11 [file peerj-cs-11-3003-s011.png]

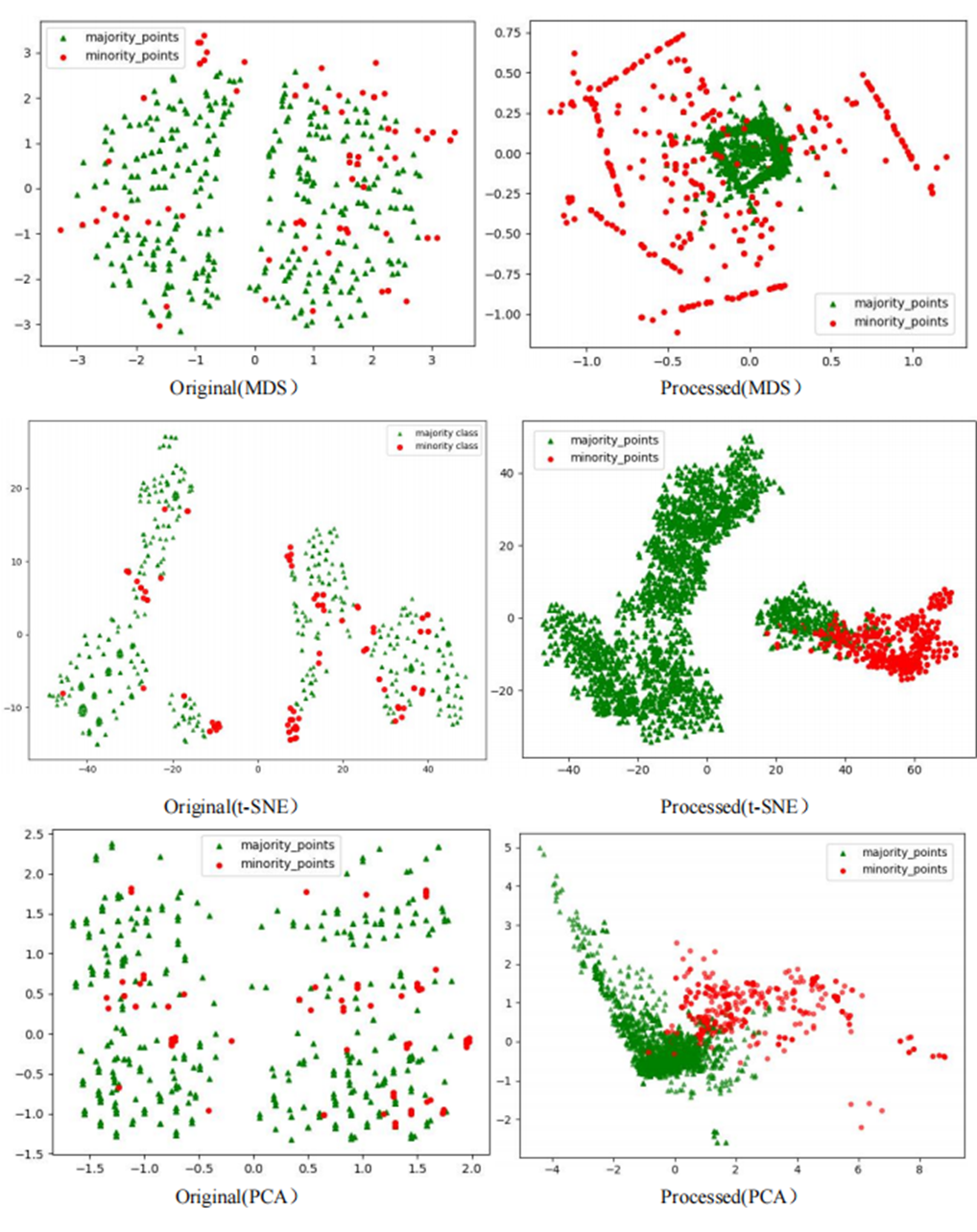

Supplement: Supplemental Information 12 [file peerj-cs-11-3003-s012.png]

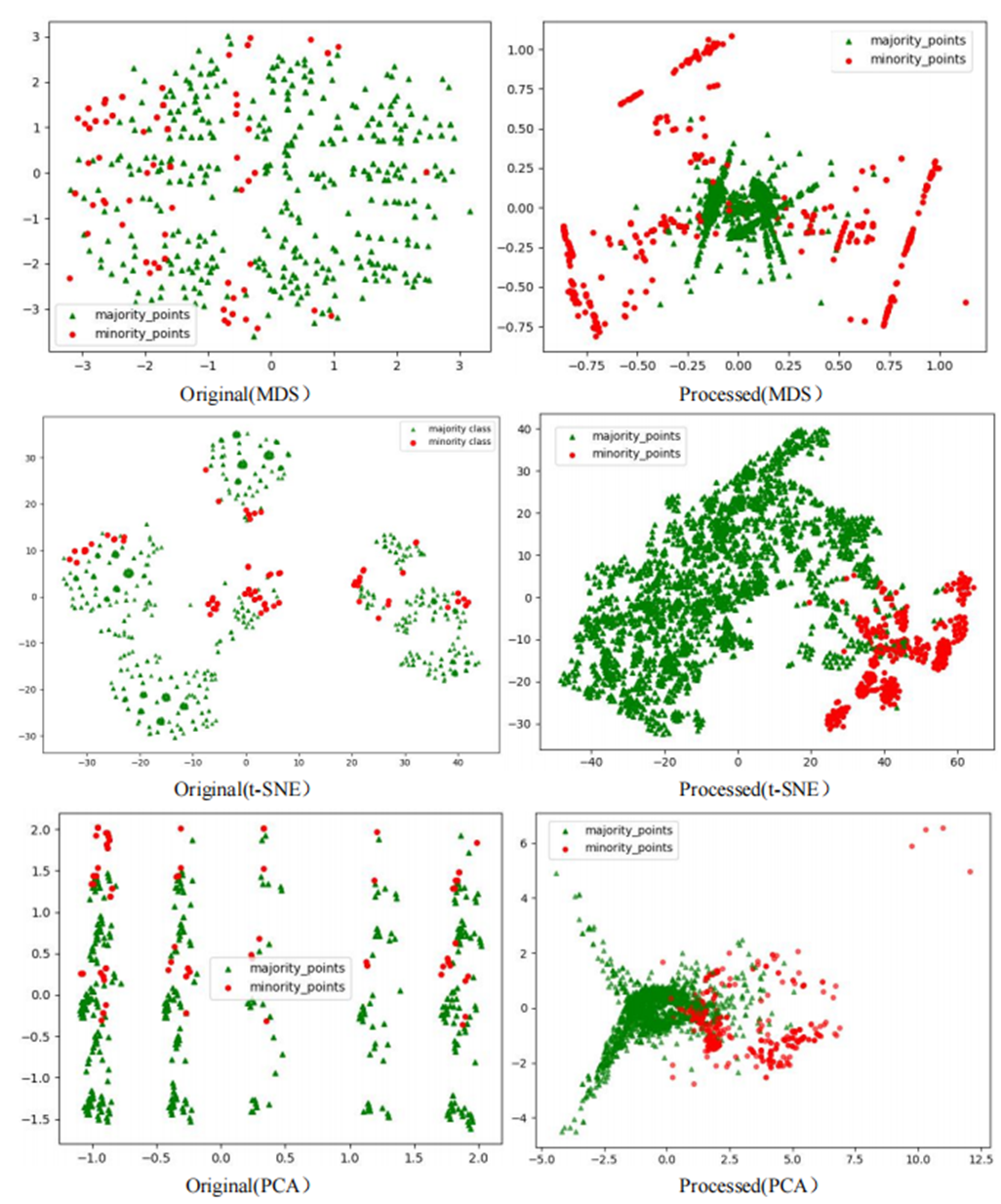

Supplement: Supplemental Information 13 [file peerj-cs-11-3003-s013.png]

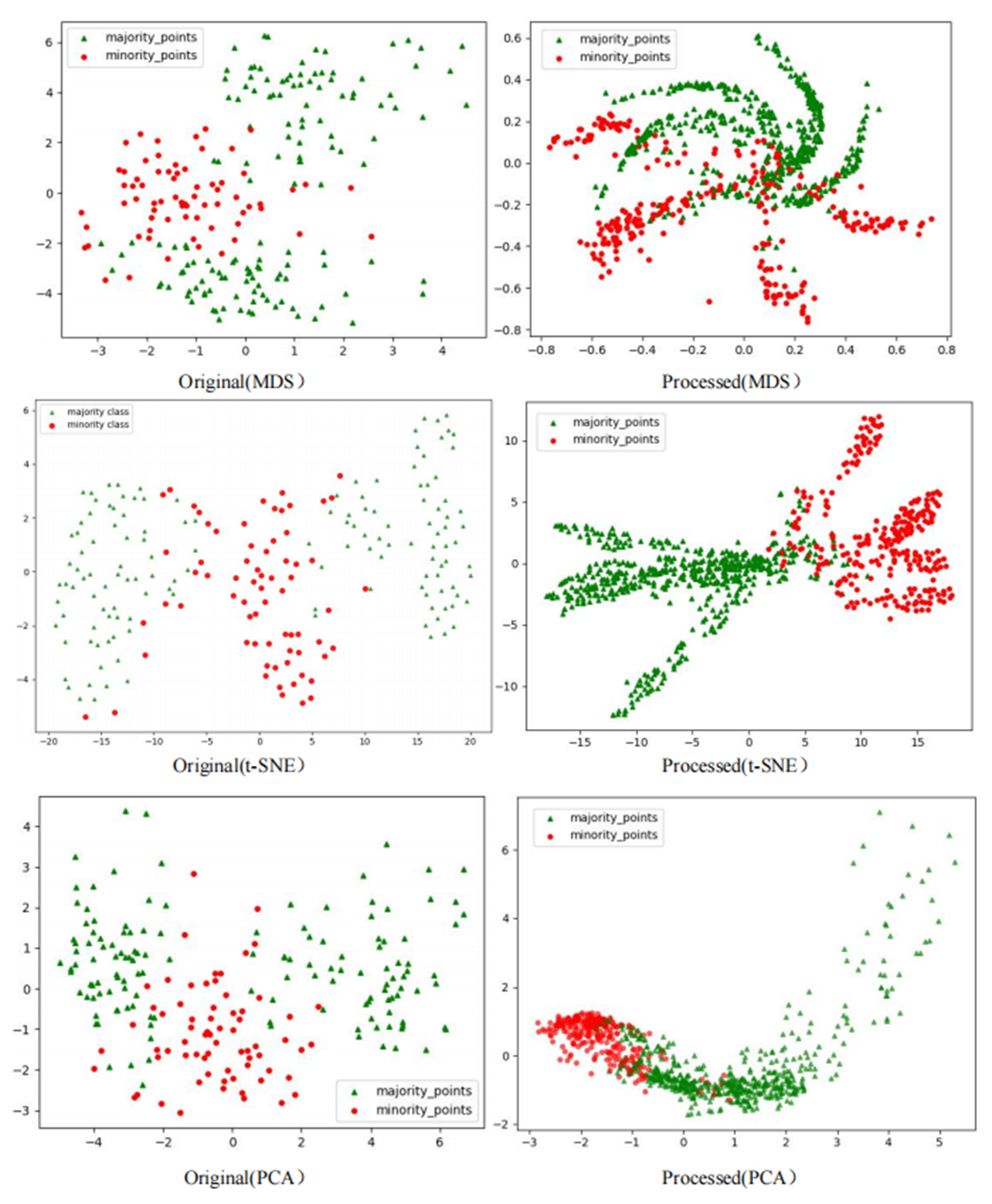

Supplement: Supplemental Information 14 [file peerj-cs-11-3003-s014.png]

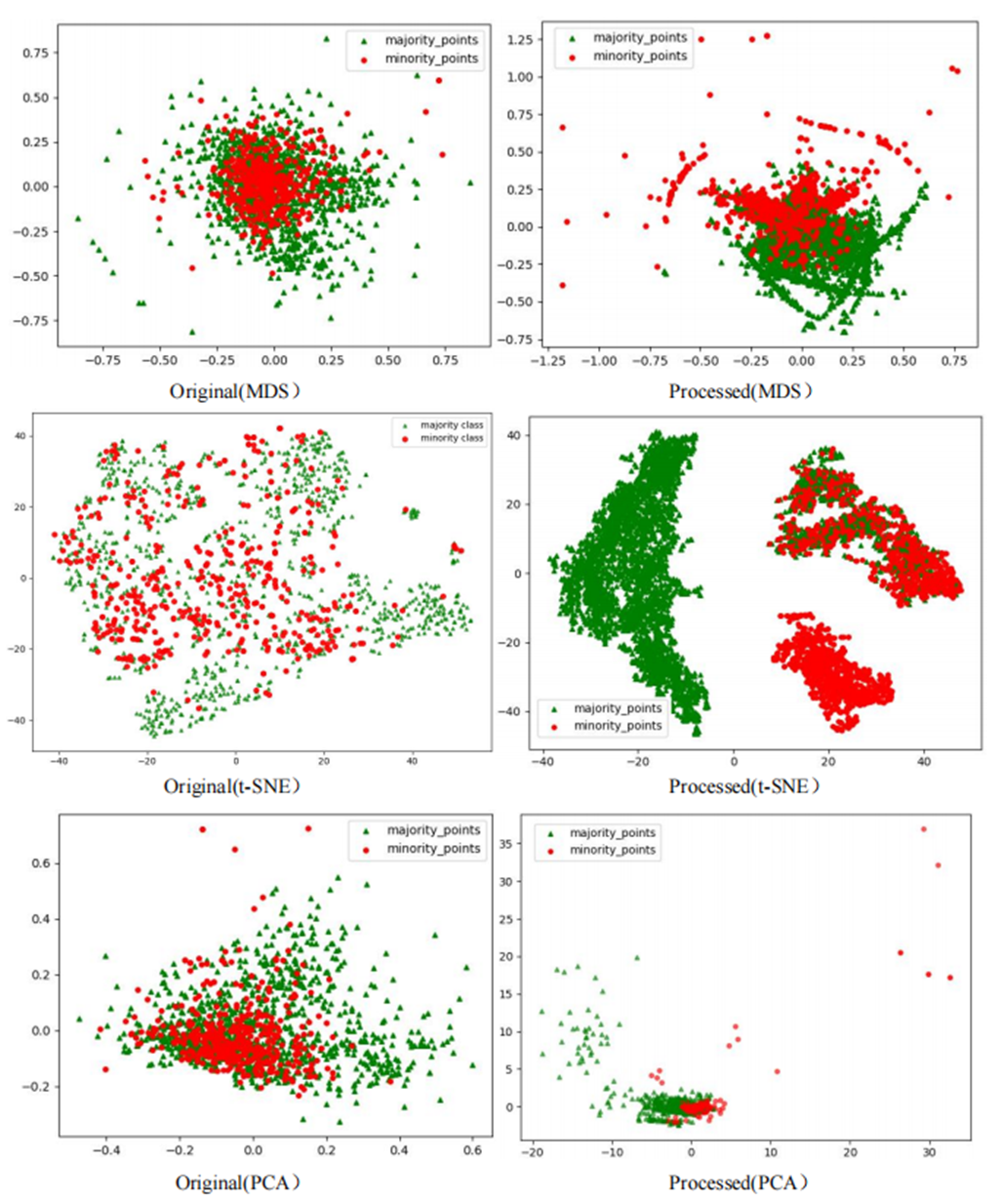

Supplement: Supplemental Information 15 [file peerj-cs-11-3003-s015.png]

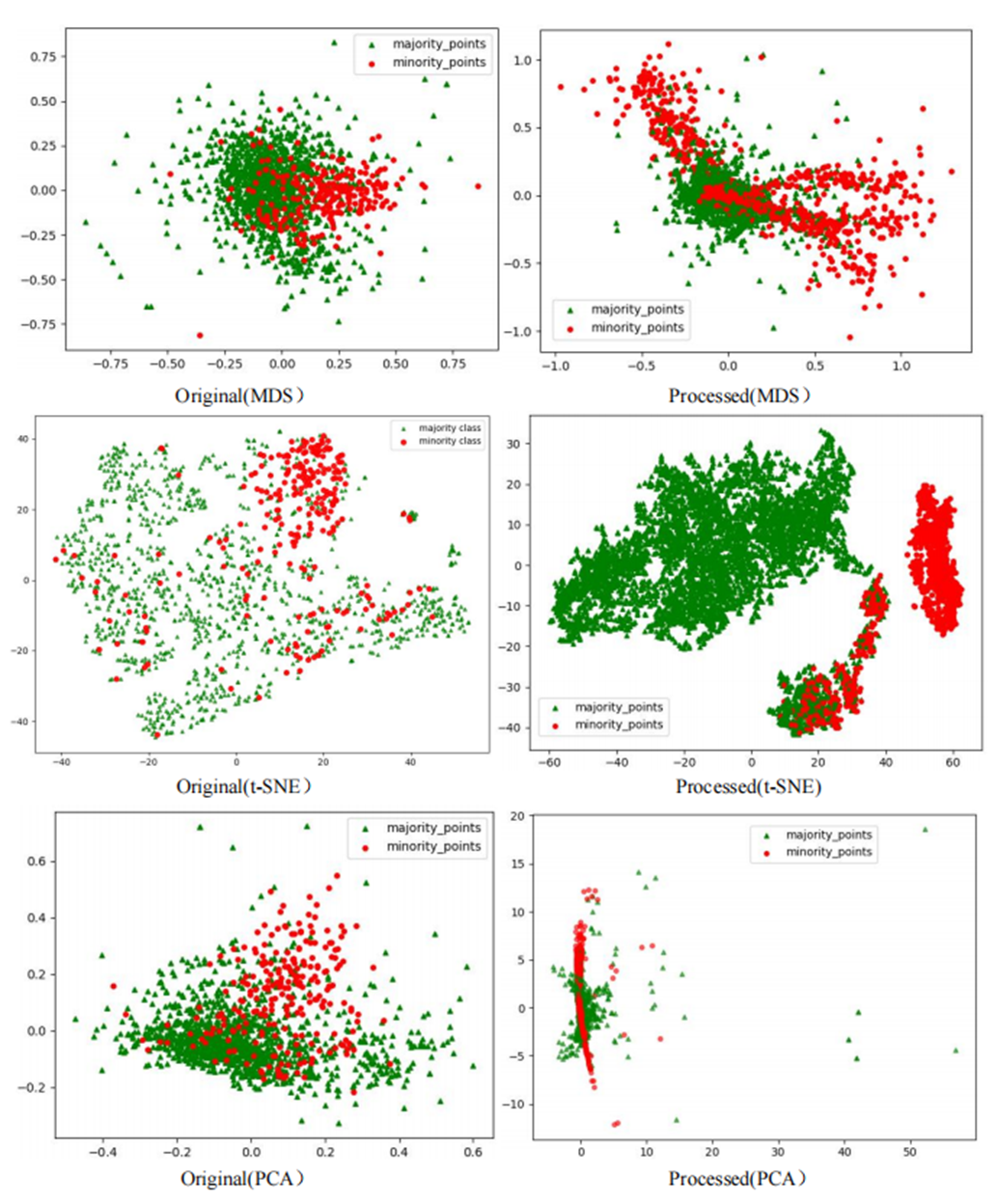

Supplement: Supplemental Information 16 [file peerj-cs-11-3003-s016.png]

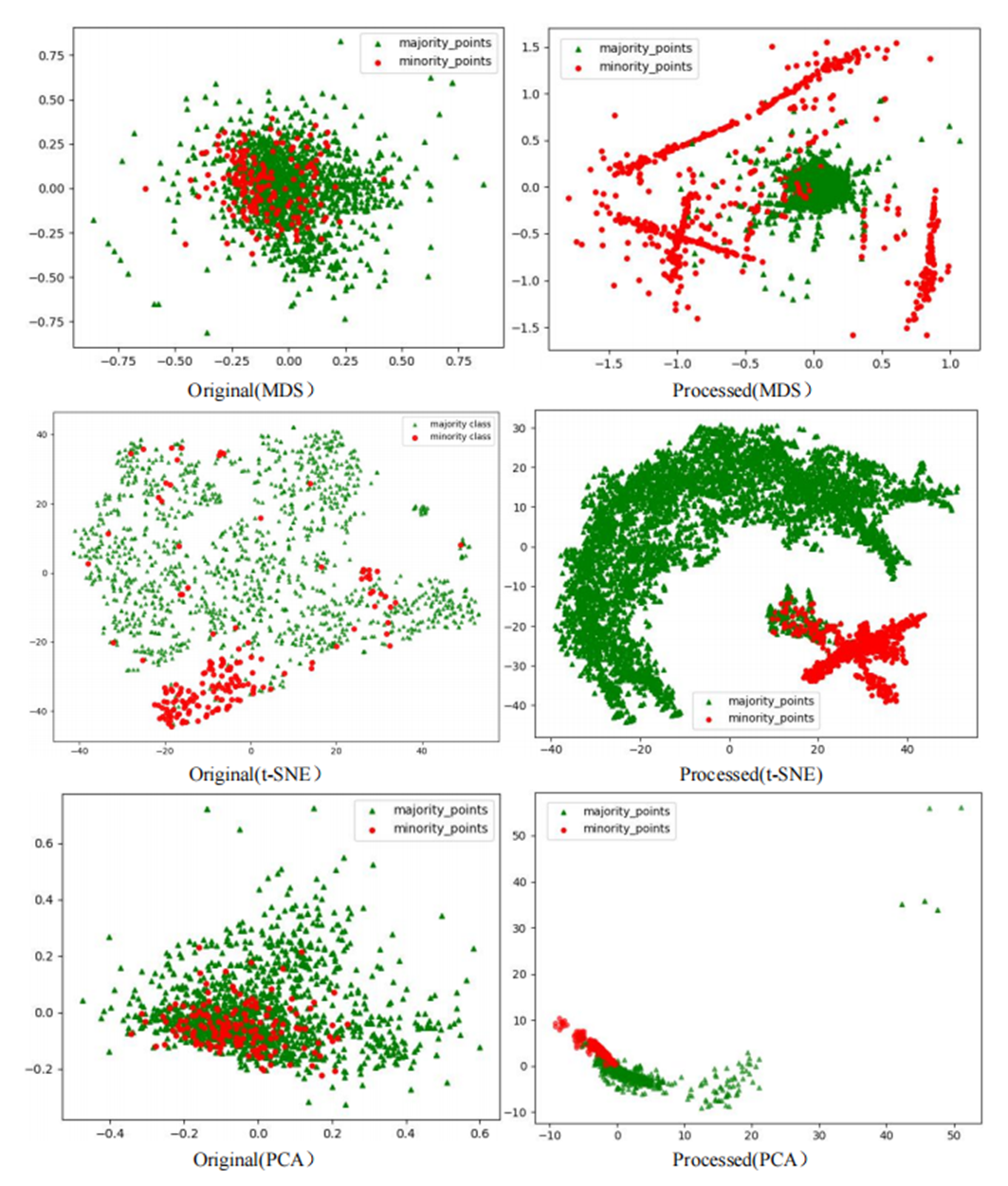

Supplement: Supplemental Information 17 [file peerj-cs-11-3003-s017.png]

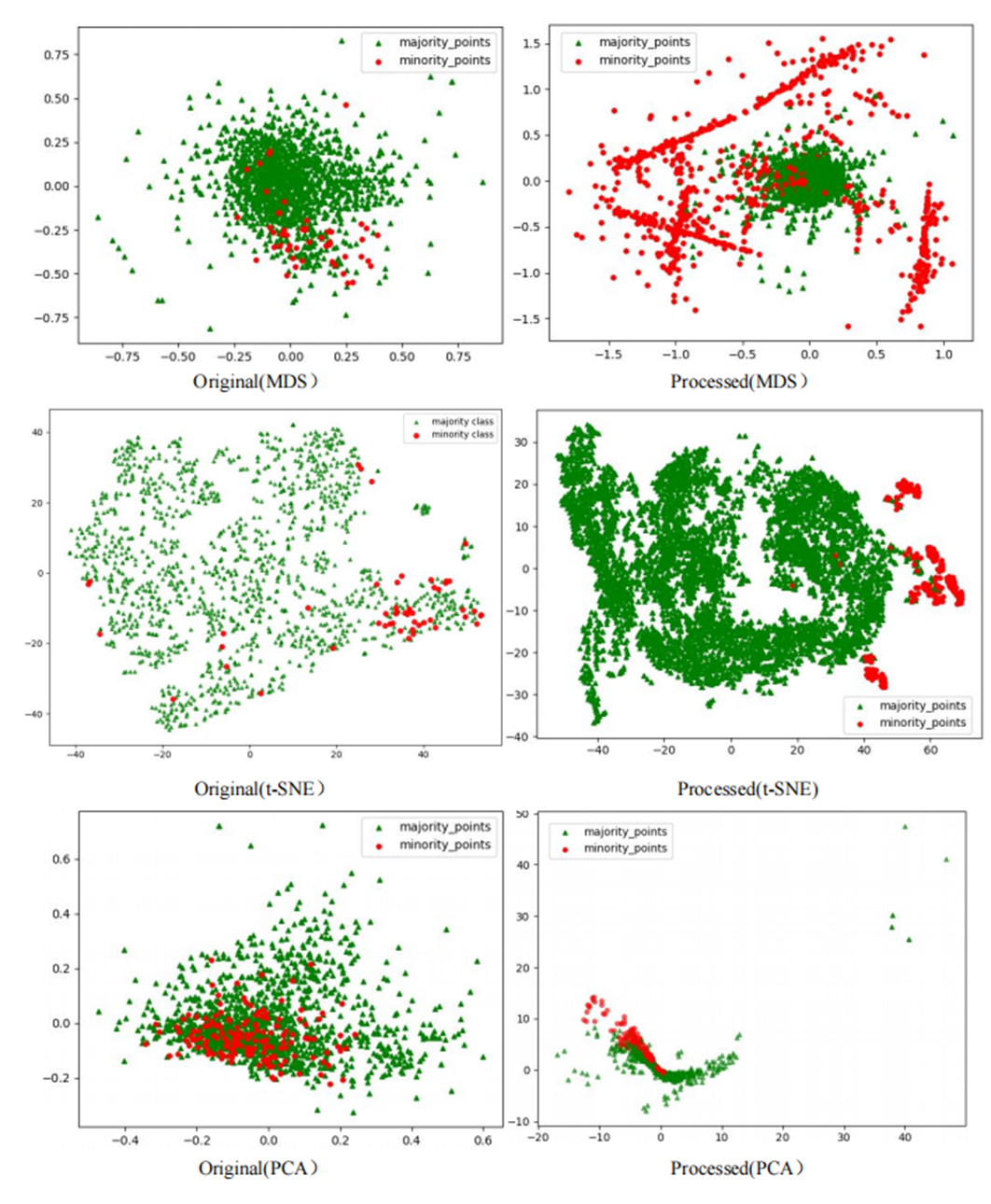

Supplement: Supplemental Information 18 [file peerj-cs-11-3003-s018.png]

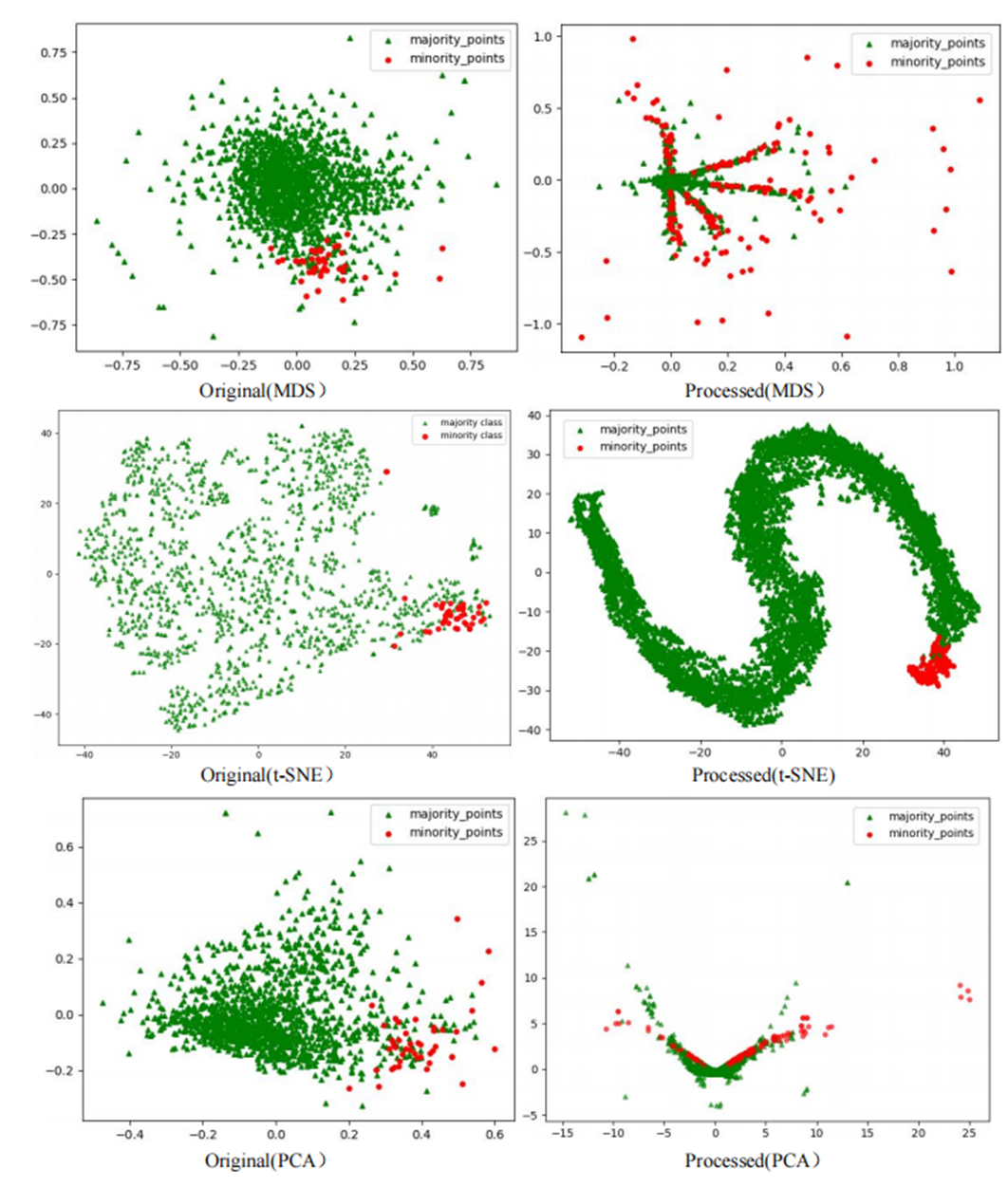

Supplement: Supplemental Information 19 [file peerj-cs-11-3003-s019.png]

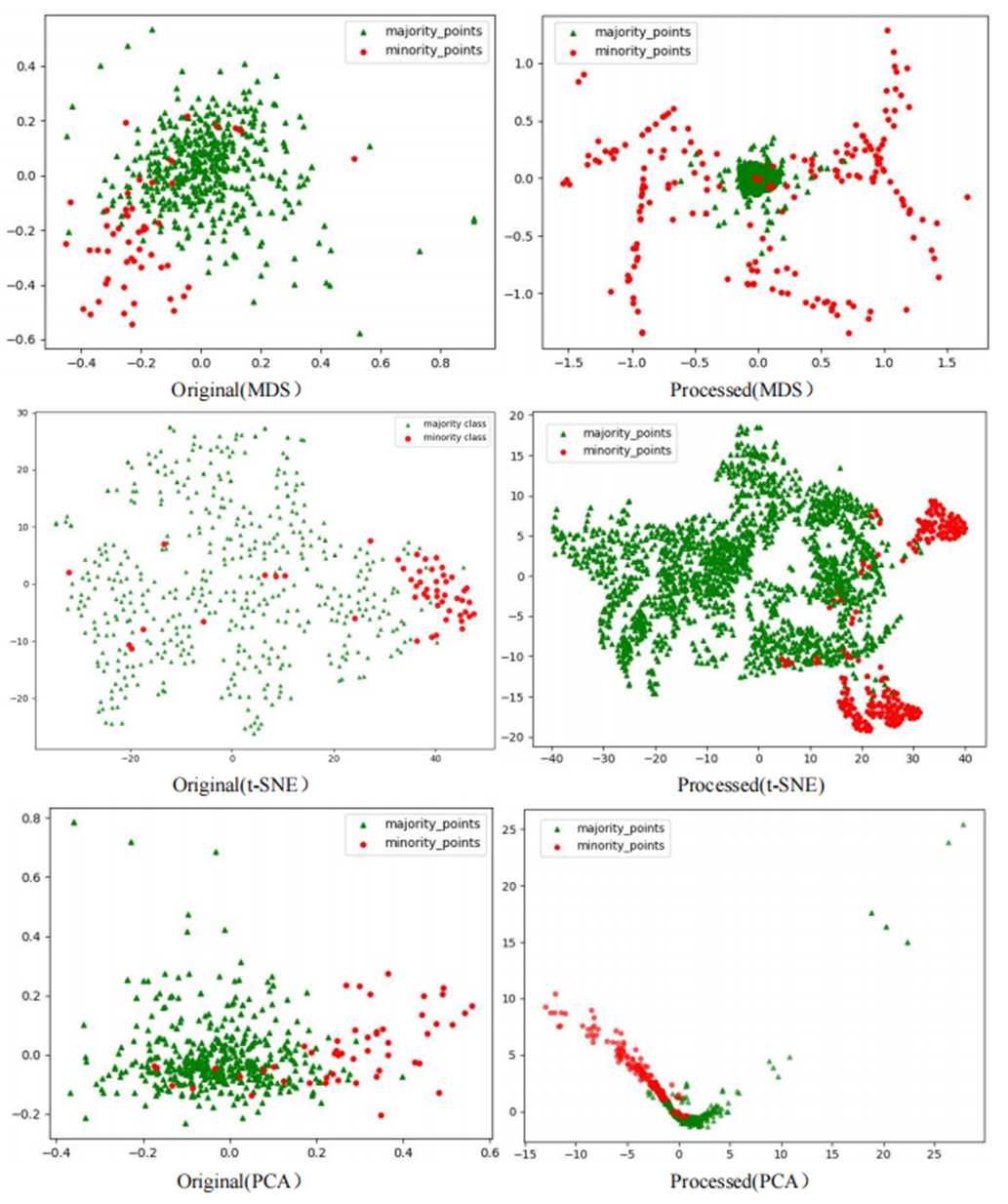

Supplement: Supplemental Information 20 [file peerj-cs-11-3003-s020.png]
