# Supplementary material for: Improving Gaussian Naive Bayes classification on imbalanced data through coordinate-based minority feature mining
Source: PeerJ Comput Sci. 2025 Jul 3;11:e3003. doi: 10.7717/peerj-cs.3003 (PMC12453872; doi:10.7717/peerj-cs.3003)
Supplement: Supplemental Information 21 [file peerj-cs-11-3003-s021.docx]

**Table S1: Classification Performance of Each Algorithm on Each Dataset When Using the GNB Model**

| Datasets | Ecoli | | | Glass1 | | |
| --- | --- | --- | --- | --- | --- | --- |
| Indicators | AUC | G-mean | F-measure | AUC | G-mean | F-measure |
| RLDC | **0.8974** | **0.8947** | **0.6257** | **0.7259** | 0.6712 | **0.6400** |
| Raw Data | 0.8250 | 0.8165 | 0.4680 | 0.7000 | 0.6735 | 0.6137 |
| BSMOTE1 | 0.8606 | 0.8541 | 0.5225 | 0.6677 | 0.6503 | 0.5819 |
| BSMOTE2 | 0.8219 | 0.8124 | 0.4310 | 0.6714 | 0.6520 | 0.5865 |
| ADASYN | 0.8627 | 0.8555 | 0.5120 | 0.6931 | 0.6637 | 0.6082 |
| SVMSMOTE | 0.8575 | 0.8506 | 0.4944 | 0.7037 | 0.6732 | 0.6176 |
| MWMOTE | 0.7937 | 0.7529 | 0.5540 | 0.7037 | 0.6738 | 0.6178 |
| KSMOTE | 0.8627 | 0.8585 | 0.4917 | 0.6963 | 0.6712 | 0.6084 |
| DPCSMOTE | 0.8603 | 0.8552 | 0.4837 | 0.7214 | 0.6898 | 0.6344 |
| SMOTE | 0.8586 | 0.8530 | 0.4851 | 0.7180 | 0.6846 | 0.6316 |
| LDBSMOTE | 0.8685 | 0.8642 | 0.5156 | 0.7256 | 0.6933 | 0.6372 |
| IBSM | 0.8735 | 0.8692 | 0.5292 | 0.7050 | 0.6763 | 0.6189 |
| SMOTETomek | 0.8685 | 0.8635 | 0.5172 | 0.7249 | **0.6945** | 0.6374 |
| SMOTEEENN | 0.8602 | 0.8546 | 0.4949 | 0.7143 | 0.6836 | 0.6276 |
| TomekLinks | 0.8393 | 0.8321 | 0.4842 | 0.7000 | 0.6735 | 0.6137 |
| AllKNN | 0.8183 | 0.8106 | 0.4430 | 0.7074 | 0.6703 | 0.6222 |
| Datasets | Glass2 | | | Haberman | | |
| Indicators | AUC | G-mean | F-measure | AUC | G-mean | F-measure |
| RLDC | **0.9446** | **0.9444** | 0.8634 | **0.6538** | **0.6085** | **0.4742** |
| Raw Data | 0.8896 | 0.8798 | 0.8444 | 0.5752 | 0.4291 | 0.2972 |
| BSMOTE1 | 0.0000 | 0.0000 | 0.0000 | 0.6029 | 0.5235 | 0.3864 |
| BSMOTE2 | 0.8379 | 0.8326 | 0.7079 | 0.6210 | 0.5664 | 0.4313 |
| ADASYN | 0.8252 | 0.8230 | 0.6749 | 0.6093 | 0.5475 | 0.4039 |
| SVMSMOTE | 0.8532 | 0.8496 | 0.7314 | 0.6079 | 0.5537 | 0.4092 |
| MWMOTE | 0.9061 | 0.9024 | 0.8478 | 0.0000 | 0.0000 | 0.0000 |
| KSMOTE | 0.8975 | 0.8922 | 0.8503 | 0.6093 | 0.5475 | 0.4039 |
| DPCSMOTE | 0.8579 | 0.8281 | 0.7941 | 0.5994 | 0.5403 | 0.3934 |
| SMOTE | 0.8925 | 0.8764 | 0.8451 | 0.6079 | 0.5537 | 0.4092 |
| LDBSMOTE | 0.9050 | 0.8953 | 0.8693 | 0.6187 | 0.5444 | 0.4127 |
| IBSM | 0.8525 | 0.8473 | 0.7412 | 0.6246 | 0.5578 | 0.4264 |
| SMOTETomek | 0.9014 | 0.8924 | 0.8640 | 0.6210 | 0.5664 | 0.4313 |
| SMOTEEENN | 0.9005 | 0.8725 | **0.8891** | 0.5875 | 0.4854 | 0.3386 |
| TomekLinks | 0.9089 | 0.9013 | 0.8518 | 0.6029 | 0.5235 | 0.3864 |
| AllKNN | 0.8993 | 0.8907 | 0.8489 | 0.6119 | 0.5207 | 0.3931 |
| Datasets | Iris | | | Letter-recognition1 | | |
| Indicators | AUC | G-mean | F-measure | AUC | G-mean | F-measure |
| RLDC | **0.9450** | **0.9432** | **0.9019** | **0.9648** | **0.9646** | **0.7454** |
| Raw Data | 0.9250 | 0.9242 | 0.8999 | 0.7098 | 0.6662 | 0.4628 |
| BSMOTE1 | 0.8678 | 0.8648 | 0.7568 | 0.5748 | 0.5430 | 0.1837 |
| BSMOTE2 | 0.8950 | 0.8943 | 0.8433 | 0.5884 | 0.5643 | 0.1940 |
| ADASYN | 0.8579 | 0.8544 | 0.7598 | 0.5824 | 0.5555 | 0.1895 |
| SVMSMOTE | 0.8605 | 0.8591 | 0.8007 | 0.6545 | 0.6518 | 0.2378 |
| MWMOTE | 0.8750 | 0.8689 | 0.8326 | 0.7911 | 0.7871 | 0.3298 |
| KSMOTE | 0.8878 | 0.8814 | 0.8437 | 0.7083 | 0.6629 | 0.4605 |
| DPCSMOTE | 0.9109 | 0.9077 | 0.8642 | 0.7759 | 0.7745 | 0.3539 |
| SMOTE | 0.9278 | 0.9267 | 0.8872 | 0.7739 | 0.7732 | 0.3611 |
| LDBSMOTE | 0.8650 | 0.8637 | 0.8065 | 0.5537 | 0.5118 | 0.1655 |
| IBSM | 0.9200 | 0.9194 | 0.8905 | 0.7482 | 0.7478 | 0.3573 |
| SMOTETomek | 0.9050 | 0.9041 | 0.8713 | 0.7727 | 0.7721 | 0.3600 |
| SMOTEEENN | 0.9250 | 0.9242 | 0.8998 | 0.7756 | 0.7754 | 0.3583 |
| TomekLinks | 0.9250 | 0.9242 | 0.8999 | 0.7100 | 0.6666 | 0.3327 |
| AllKNN | 0.9200 | 0.9194 | 0.8905 | 0.7110 | 0.6689 | 0.3394 |
| Datasets | Letter-recognition2 | | | Letter-recognition3 | | |
| Indicators | AUC | G-mean | F-measure | AUC | G-mean | F-measure |
| RLDC | **0.9563** | **0.9561** | **0.8392** | **0.9684** | **0.9682** | **0.6044** |
| Raw Data | 0.7079 | 0.6809 | 0.5006 | 0.8294 | 0.8242 | 0.3968 |
| BSMOTE1 | 0.6165 | 0.6163 | 0.3312 | 0.6603 | 0.6514 | 0.1498 |
| BSMOTE2 | 0.6248 | 0.6246 | 0.3389 | 0.6680 | 0.6618 | 0.1510 |
| ADASYN | 0.6438 | 0.6427 | 0.3549 | 0.7152 | 0.7141 | 0.1719 |
| SVMSMOTE | 0.6907 | 0.6822 | 0.3909 | 0.8347 | 0.8240 | 0.2062 |
| MWMOTE | 0.7220 | 0.7143 | 0.4211 | 0.8235 | 0.8223 | 0.2383 |
| KSMOTE | 0.6512 | 0.6392 | 0.3782 | 0.7603 | 0.7515 | 0.2682 |
| DPCSMOTE | 0.7276 | 0.7236 | 0.4327 | 0.8184 | 0.8178 | 0.2421 |
| SMOTE | 0.7124 | 0.7081 | 0.4166 | 0.8136 | 0.8126 | 0.2295 |
| LDBSMOTE | 0.6165 | 0.6160 | 0.3323 | 0.5787 | 0.5423 | 0.1102 |
| IBSM | 0.7197 | 0.7157 | 0.4350 | 0.8253 | 0.8139 | 0.2366 |
| SMOTETomek | 0.7132 | 0.7090 | 0.4175 | 0.8123 | 0.8113 | 0.2281 |
| SMOTEEENN | 0.7151 | 0.7094 | 0.4170 | 0.8128 | 0.8116 | 0.2274 |
| TomekLinks | 0.7082 | 0.6814 | 0.5008 | 0.8294 | 0.8242 | 0.2367 |
| AllKNN | 0.7131 | 0.6897 | 0.5037 | 0.8315 | 0.8265 | 0.2361 |
| Datasets | Poker-hand1 | | | Poker-hand2 | | |
| Indicators | AUC | G-mean | F-measure | AUC | G-mean | F-measure |
| RLDC | **0.5929** | 0.4749 | **0.2553** | **0.5563** | 0.3865 | **0.1544** |
| Raw Data | 0.5000 | 0.0000 | NaN | 0.5000 | 0.0000 | NaN |
| BSMOTE1 | 0.4895 | 0.4701 | 0.1206 | 0.4830 | 0.4584 | 0.0776 |
| BSMOTE2 | 0.4911 | 0.4582 | 0.1183 | 0.4818 | 0.4442 | 0.0753 |
| ADASYN | 0.4884 | 0.4654 | 0.1191 | 0.4810 | 0.4523 | 0.0761 |
| SVMSMOTE | 0.4926 | 0.4742 | 0.1226 | 0.5007 | 0.3712 | 0.0736 |
| MWMOTE | 0.4970 | 0.4752 | 0.1274 | 0.5012 | 0.0440 | 0.0109 |
| KSMOTE | 0.5087 | 0.4683 | 0.1285 | 0.5000 | 0.4567 | 0.0827 |
| DPCSMOTE | 0.4946 | 0.4816 | 0.1251 | 0.4936 | 0.4764 | 0.0827 |
| SMOTE | 0.4863 | 0.4648 | 0.1183 | 0.4847 | 0.4574 | 0.0779 |
| LDBSMOTE | 0.5019 | **0.4904** | 0.1295 | 0.5087 | **0.4909** | 0.0888 |
| IBSM | 0.4979 | 0.4863 | 0.1272 | 0.4934 | 0.4769 | 0.0827 |
| SMOTETomek | 0.4881 | 0.4673 | 0.1194 | 0.4843 | 0.4573 | 0.0777 |
| SMOTEEENN | 0.4946 | 0.4883 | 0.1332 | 0.4837 | 0.4823 | 0.0829 |
| TomekLinks | 0.5000 | 0.0000 | 0.0000 | 0.5000 | 0.0000 | nan |
| AllKNN | 0.5000 | 0.0000 | 0.0000 | 0.5000 | 0.0000 | nan |
| Datasets | Phishingdata1 | | | Phishingdata2 | | |
| Indicators | AUC | G-mean | F-measure | AUC | G-mean | F-measure |
| RLDC | **0.8992** | **0.8984** | **0.6322** | **0.8964** | **0.8961** | **0.7369** |
| Raw Data | 0.5824 | 0.3960 | 0.2667 | 0.7259 | 0.6909 | 0.5547 |
| BSMOTE1 | 0.6598 | 0.6501 | 0.2178 | 0.7342 | 0.7330 | 0.4665 |
| BSMOTE2 | 0.6599 | 0.6508 | 0.2197 | 0.7299 | 0.7290 | 0.4568 |
| ADASYN | 0.6419 | 0.6346 | 0.2088 | 0.7409 | 0.7403 | 0.4684 |
| SVMSMOTE | 0.6129 | 0.6006 | 0.2024 | 0.7643 | 0.7632 | 0.5140 |
| MWMOTE | 0.6495 | 0.6412 | 0.2222 | 0.7341 | 0.7271 | 0.4858 |
| KSMOTE | 0.6864 | 0.6817 | 0.2477 | 0.7616 | 0.7573 | 0.5324 |
| DPCSMOTE | 0.6715 | 0.6648 | 0.2361 | 0.7528 | 0.7477 | 0.5103 |
| SMOTE | 0.6369 | 0.6288 | 0.2070 | 0.7426 | 0.7378 | 0.5049 |
| LDBSMOTE | 0.6987 | 0.6502 | 0.2430 | 0.7651 | 0.7624 | 0.5200 |
| IBSM | 0.6862 | 0.6584 | 0.2428 | 0.7521 | 0.7477 | 0.5048 |
| SMOTETomek | 0.6493 | 0.6403 | 0.2168 | 0.7492 | 0.7452 | 0.5091 |
| SMOTEEENN | 0.6769 | 0.6704 | 0.2361 | 0.7323 | 0.7257 | 0.4828 |
| TomekLinks | 0.5820 | 0.3958 | 0.2648 | 0.7259 | 0.6910 | 0.5556 |
| AllKNN | 0.5976 | 0.4399 | 0.2895 | 0.7302 | 0.6992 | 0.5526 |
| Datasets | Phishingdata3 | | | Seeds | | |
| Indicators | AUC | G-mean | F-measure | AUC | G-mean | F-measure |
| RLDC | **0.9185** | **0.9171** | **0.7379** | **0.9000** | **0.8979** | **0.8481** |
| Raw Data | 0.7030 | 0.6648 | 0.4898 | 0.8750 | 0.8712 | 0.8295 |
| BSMOTE1 | 0.7546 | 0.7543 | 0.4377 | 0.8399 | 0.8348 | 0.7728 |
| BSMOTE2 | 0.7640 | 0.7578 | 0.4225 | 0.8464 | 0.8401 | 0.7835 |
| ADASYN | 0.7649 | 0.7644 | 0.4513 | 0.8468 | 0.8458 | 0.7777 |
| SVMSMOTE | 0.8062 | 0.8031 | 0.5061 | 0.8696 | 0.8649 | 0.8093 |
| MWMOTE | 0.7943 | 0.7931 | 0.5156 | 0.8179 | 0.8063 | 0.7596 |
| KSMOTE | 0.7724 | 0.7688 | 0.4933 | 0.8641 | 0.8625 | 0.8008 |
| DPCSMOTE | 0.7924 | 0.7905 | 0.5171 | 0.8517 | 0.8474 | 0.7958 |
| SMOTE | 0.8112 | 0.8084 | 0.5182 | 0.8689 | 0.8663 | 0.7960 |
| LDBSMOTE | 0.8081 | 0.8070 | 0.5229 | 0.8536 | 0.8475 | 0.7964 |
| IBSM | 0.8052 | 0.8027 | 0.5220 | 0.8321 | 0.8254 | 0.7735 |
| SMOTETomek | 0.8174 | 0.8155 | 0.5274 | 0.8714 | 0.8683 | 0.8229 |
| SMOTEEENN | 0.8093 | 0.8080 | 0.5347 | 0.8464 | 0.8318 | 0.7935 |
| TomekLinks | 0.7015 | 0.6638 | 0.4848 | 0.8679 | 0.8641 | 0.8183 |
| AllKNN | 0.7089 | 0.6748 | 0.4906 | 0.8821 | 0.8800 | 0.8361 |
| Datasets | Yeast1 | | | Yeast2 | | |
| Indicators | AUC | G-mean | F-measure | AUC | G-mean | F-measure |
| RLDC | **0.6598** | **0.6197** | 0.5148 | **0.7218** | **0.6589** | **0.4880** |
| Raw Data | 0.6375 | 0.5524 | 0.5530 | 0.5313 | 0.2891 | 0.2948 |
| BSMOTE1 | 0.6206 | 0.4985 | 0.5356 | 0.5282 | 0.2724 | 0.2802 |
| BSMOTE2 | 0.6399 | 0.5648 | 0.5542 | 0.6037 | 0.4623 | 0.3375 |
| ADASYN | 0.6104 | 0.4795 | 0.5394 | 0.5420 | 0.3178 | 0.3132 |
| SVMSMOTE | 0.6182 | 0.4955 | 0.5419 | 0.5318 | 0.2720 | 0.2956 |
| MWMOTE | 0.6372 | 0.5422 | 0.5547 | 0.5930 | 0.4629 | 0.3331 |
| KSMOTE | 0.6027 | 0.5307 | 0.5193 | 0.5803 | 0.4512 | 0.3192 |
| DPCSMOTE | 0.6340 | 0.5362 | **0.5552** | 0.5186 | 0.2170 | 0.4559 |
| SMOTE | 0.6263 | 0.5044 | 0.5503 | 0.5454 | 0.3390 | 0.2955 |
| LDBSMOTE | 0.5833 | 0.5073 | 0.4861 | 0.5138 | 0.2467 | 0.2864 |
| IBSM | 0.5773 | 0.5014 | 0.4735 | 0.5446 | 0.3313 | 0.3011 |
| SMOTETomek | 0.6062 | 0.4636 | 0.5360 | 0.5490 | 0.3445 | 0.3031 |
| SMOTEEENN | 0.5252 | 0.2707 | 0.4858 | 0.5514 | 0.3647 | 0.3041 |
| TomekLinks | 0.6378 | 0.5474 | 0.5543 | 0.5313 | 0.2891 | 0.2948 |
| AllKNN | 0.6229 | 0.5010 | 0.5458 | 0.5317 | 0.2844 | 0.2952 |
| Datasets | Yeast3 | | | Yeast4 | | |
| Indicators | AUC | G-mean | F-measure | AUC | G-mean | F-measure |
| RLDC | 0.7282 | 0.6964 | 0.2985 | **0.7867** | **0.7823** | 0.1855 |
| Raw Data | 0.6094 | 0.4676 | 0.2418 | 0.5522 | 0.3639 | 0.0743 |
| BSMOTE1 | 0.5701 | 0.3816 | 0.2199 | 0.5693 | 0.4203 | 0.0754 |
| BSMOTE2 | **0.7623** | **0.7162** | 0.3846 | 0.6437 | 0.5666 | 0.0974 |
| ADASYN | 0.5902 | 0.4306 | 0.2217 | 0.5323 | 0.3586 | 0.0700 |
| SVMSMOTE | 0.6437 | 0.5359 | 0.2601 | 0.6055 | 0.5369 | 0.0869 |
| MWMOTE | 0.7092 | 0.6446 | **0.4330** | 0.5970 | 0.4367 | **0.2199** |
| KSMOTE | 0.6381 | 0.5087 | 0.2707 | 0.4979 | 0.4475 | 0.0596 |
| DPCSMOTE | 0.6376 | 0.5317 | 0.2563 | 0.5504 | 0.3743 | 0.0723 |
| SMOTE | 0.6246 | 0.5133 | 0.2357 | 0.5710 | 0.3934 | 0.0755 |
| LDBSMOTE | 0.6064 | 0.4613 | 0.2399 | 0.5883 | 0.4428 | 0.0851 |
| IBSM | 0.6169 | 0.4932 | 0.2454 | 0.5623 | 0.3928 | 0.0759 |
| SMOTETomek | 0.6211 | 0.4975 | 0.2470 | 0.5686 | 0.4081 | 0.0770 |
| SMOTEEENN | 0.6203 | 0.4999 | 0.2470 | 0.5679 | 0.4066 | 0.0768 |
| TomekLinks | 0.6097 | 0.4683 | 0.2420 | 0.5536 | 0.3671 | 0.0746 |
| AllKNN | 0.6132 | 0.4739 | 0.2439 | 0.5609 | 0.3844 | 0.0759 |
| Datasets | Yeast5 | | | Yeast6 | | |
| Indicators | AUC | G-mean | F-measure | AUC | G-mean | F-measure |
| RLDC | **0.9528** | **0.9523** | **0.5459** | **0.8602** | **0.8550** | 0.5693 |
| Raw Data | 0.8260 | 0.8074 | 0.1498 | 0.6105 | 0.4329 | 0.2945 |
| BSMOTE1 | 0.8450 | 0.8304 | 0.1691 | 0.6171 | 0.4571 | 0.2571 |
| BSMOTE2 | 0.7368 | 0.6870 | 0.1162 | 0.6997 | 0.6382 | 0.3053 |
| ADASYN | 0.8399 | 0.8244 | 0.1643 | 0.5495 | 0.4033 | 0.2131 |
| SVMSMOTE | 0.8257 | 0.8069 | 0.1501 | 0.5906 | 0.3580 | 0.2677 |
| MWMOTE | 0.6111 | 0.4695 | 0.3291 | 0.7598 | 0.7254 | 0.5588 |
| KSMOTE | 0.8450 | 0.8340 | 0.1998 | 0.5868 | 0.5339 | 0.2417 |
| DPCSMOTE | 0.8837 | 0.8748 | 0.2338 | 0.5497 | 0.3815 | 0.2384 |
| SMOTE | 0.8674 | 0.8565 | 0.1970 | 0.5840 | 0.4243 | 0.2343 |
| LDBSMOTE | 0.8701 | 0.8631 | 0.2038 | 0.6209 | 0.4692 | 0.2661 |
| IBSM | 0.8635 | 0.8526 | 0.1834 | 0.6274 | 0.4694 | 0.2858 |
| SMOTETomek | 0.8681 | 0.8579 | 0.1890 | 0.6288 | 0.5046 | 0.2887 |
| SMOTEEENN | 0.8674 | 0.8571 | 0.1883 | 0.7614 | 0.7110 | 0.5145 |
| TomekLinks | 0.8260 | 0.8074 | 0.1498 | 0.6138 | 0.4399 | 0.2960 |
| AllKNN | 0.8260 | 0.8074 | 0.1498 | 0.7534 | 0.6557 | **0.5705** |
