# Supplementary material for: Improving Gaussian Naive Bayes classification on imbalanced data through coordinate-based minority feature mining
Source: PeerJ Comput Sci. 2025 Jul 3;11:e3003. doi: 10.7717/peerj-cs.3003 (PMC12453872; doi:10.7717/peerj-cs.3003)
Supplement: Supplemental Information 22 [file peerj-cs-11-3003-s022.docx]

**Table S2: Experimental parameter setting of the RLDC algorithm**

| Dataset | The number T of division intercepts. |
| --- | --- |
| M_DATA2 | 36 |
| Ecoli | 6 |
| Glass1 | 6 |
| Glass2 | 8 |
| Haberman | 33 |
| Iris | 9 |
| Letter-recognition1 | 10 |
| Letter-recognition2 | 10 |
| Letter-recognition3 | 10 |
| Poker-hand1 | 5 |
| Poker-hand2 | 5 |
| Phishingdata1 | 5 |
| Phishingdata2 | 5 |
| Phishingdata3 | 5 |
| Seeds | 6 |
| Yeast1 | 9 |
| Yeast2 | 9 |
| Yeast3 | 23 |
| Yeast4 | 14 |
| Yeast5 | 6 |
| Yeast6 | 10 |
