# Supplementary material for: Improving Gaussian Naive Bayes classification on imbalanced data through coordinate-based minority feature mining
Source: PeerJ Comput Sci. 2025 Jul 3;11:e3003. doi: 10.7717/peerj-cs.3003 (PMC12453872; doi:10.7717/peerj-cs.3003)
Supplement: Supplemental Information 23 [file peerj-cs-11-3003-s023.docx]

**Table S3 Results of the Scalability Experiments​**

| SampleSize | Number of Attributes | ImbalanceRatio | Time(s) | Memory(MB) |
| --- | --- | --- | --- | --- |
| 1000 | 10 | 3 | 0.11 | 205.30 |
| 1000 | 50 | 3 | 0.17 | 205.70 |
| 1000 | 100 | 3 | 0.25 | 207.30 |
| 1000 | 200 | 3 | 0.39 | 210.90 |
| 5000 | 10 | 3 | 0.92 | 209.70 |
| 5000 | 50 | 3 | 2.25 | 211.70 |
| 5000 | 100 | 3 | 4.16 | 215.30 |
| 5000 | 200 | 3 | 7.83 | 223.00 |
| 10000 | 10 | 3 | 2.86 | 216.10 |
| 10000 | 50 | 3 | 7.98 | 218.30 |
| 10000 | 100 | 3 | 16.04 | 223.20 |
| 10000 | 200 | 3 | 37.35 | 235.80 |
| 100000 | 10 | 3 | 229.67 | 219.00 |
| 100000 | 50 | 3 | 1531.49 | 250.60 |
| 100000 | 100 | 3 | 3992.78 | 291.00 |
| 100000 | 200 | 3 | 6277.49 | 371.50 |
| 1000 | 10 | 10 | 0.10 | 198.80 |
| 1000 | 50 | 10 | 0.16 | 201.60 |
| 1000 | 100 | 10 | 0.23 | 204.30 |
| 1000 | 200 | 10 | 0.38 | 206.00 |
| 5000 | 10 | 10 | 0.89 | 206.30 |
| 5000 | 50 | 10 | 2.20 | 209.80 |
| 5000 | 100 | 10 | 3.78 | 213.50 |
| 5000 | 200 | 10 | 7.37 | 221.90 |
| 10000 | 10 | 10 | 2.66 | 215.50 |
| 10000 | 50 | 10 | 7.27 | 219.30 |
| 10000 | 100 | 10 | 14.95 | 221.90 |
| 10000 | 200 | 10 | 32.64 | 233.00 |
| 100000 | 10 | 10 | 161.95 | 230.50 |
| 100000 | 50 | 10 | 1400.85 | 270.10 |
| 100000 | 100 | 10 | 3366.91 | 312.40 |
| 100000 | 200 | 10 | 9504.32 | 372.10 |
